# Intragenic antimicrobial peptides from *Theobroma cacao*

**DOI:** 10.1186/1753-6561-8-S4-P106

**Published:** 2014-10-01

**Authors:** Marcelo Ramada, Guilherme Brand, Fernando Abrão, Lucia Souza, Maria Silva, Carlos Bloch

**Affiliations:** 1 EMBRAPA Recursos Genéticos e Biotecnologia - Parque Estação Biológica, 70770-917, Brasília-DF, Brazil; 2 Instituto de Patologia Tropical e Saúde Pública (IPTSP), Universidade Federal de Goiás, 74605-050, Goiânia-GO, Brazil

## Background

It is well known that many bioactive peptides (intragenic) are encrypted in source proteins and that they can exert their function once released by proteolytic cleavage; e.g. hypotensive, opioids and antimicrobial peptides. However, other bioactive peptides may be "stuck" on a polipeptide chain with no cleavage sites for its release. These "non-obvious" intragenic peptides are also of interest in the search for new biologically active peptides, mainly antimicrobial peptides, in an alternative way for new drug discovery and for the control of different phytopathogens, mainly fungi, that can cause several losses to different crops of interest; e.g. rice, soybean, common bean, cocoa. In Brazil, *Theobroma cacao *production can be decimate by the basidiomycete *Moniliophtora perniciosa*, the causative agent of cocoa witch's broom disease. In this report we present preliminary results of the search, synthesis and activity of intragenic antimicrobial peptides (IAPs) selected from *Theobroma cacao *genome.

## Methods

In this study, we performed a search of putative IAPs using *Theobroma cacao *Matina 1.6 genome [1]. Search was performed using the software Kamal v1.0 alpha [2] with user-created parameters. Eleven peptides out of 700000 filtered peptides were selected for in-house solid-phase synthesis. DS01 [3] and Ascaphin-8 [4] were also synthesized as positive controls. Peptides were purified by RP-HPLC in a preparative C18 column. The purity and molecular mass of peptides were evaluated by MALDI-TOF MS (UltraFlex III, Bruker Daltonics). Peptide fragmentation was obtained by MALDI-TOF MS/MS experiments and the resulting data were analyzed manually using Flex Analysis 3.0 (Bruker Daltonics) software to confirm synthetic peptides aminoacid sequence. The minimum inhibitory concentrations (MIC) of the synthetic peptides for *Candida albicans *ATCC 90028 and *Cryptococcus neoformans *ATCC 28957 was determined by microdilution broth method, according to CLSI M27-3a document [5] and were evaluated at concentrations between 256 μM-0.5 μM.

## Results and conclusions

Pep2, Pep5, Pep6, Pep8 and Pep10 showed MICs against *C. neoformans *ATCC and *C. albicans *ATCC. The data obtained for DS01 and Ascaphin-8 for *C. albicans *showed MICs of 6 and 8 μM, respectively, in agreement with the literature [3,4]. Pep5 and Pep10 showed MICs of 128 and 64 μM, respectively. Pep6 and Pep8 inhibit *C. albicans *and *C. neoformans *growth at 4 μM and 8 μM. Pep2 was able to inhibit and kill both fungi at 2 μM. Pep2, 6 and 8 showed lower MIC values than DS01 and Ascaphin-8 for *C. neoformans*. Synthetic peptides 2, 6 and 8 showed promising results against human pathogenic fungi highlighting the importance of this approach to search for new drugs. To evaluate if this approach can render promising results for agriculture, which is the main goal of our study, MICs assays are being performed with some fungi phytopathogens. This approach can be use as an alternative to the transgenic technology as the plant own information, not an exogenous one, could be used for the control of phytopathogens, as proposed for soybean [2].
